# Quantitative Detection of Key Parameters and Authenticity Verification for Beer Using Near-Infrared Spectroscopy

**DOI:** 10.3390/foods14223936

**Published:** 2025-11-17

**Authors:** Yongshun Wei, Jinming Liu, Guiqing Xi, Yuhao Lu

**Affiliations:** College of Information and Electrical Engineering, Heilongjiang Bayi Agricultural University, Daqing 163319, China; weiyongshun@byau.edu.cn (Y.W.); xiguiqing@byau.edu.cn (G.X.); luyuhao@byau.edu.cn (Y.L.)

**Keywords:** beer, alcohol content, original wort concentration, authenticity verification, near-infrared spectroscopy

## Abstract

Alcohol content and original wort concentration are key indicators of beer quality. The detection of these metrics and the authentication of beer authenticity are crucial for protecting consumer rights. To this end, this study investigates quantitative detection methods for beer alcohol content and original wort concentration based on near-infrared spectroscopy (NIRS), as well as authenticity verification methods for craft, industrial, and non-fermented beers. Convolutional neural networks combined with a long short-term memory networks (CNN-LSTM) feature extraction method was proposed for establishing multiple regression models and partial least squares discriminant analysis (PLS-DA) model. The results indicate that the CNN-LSTM combined with the support vector machine regression demonstrates optimal performance, with coefficients of determination exceeding 0.99 for the alcohol content calibration, validation, and independent test sets, and all relative root mean square errors below 2.67%. For original wort concentration, the coefficients of determination exceeded 0.97 across the calibration, validation, and independent test sets, with relative root mean square errors below 4.05%. The CNN-LSTM combined with the PLS-DA approach exhibited the lowest variable dimension while achieving 100% classification accuracy. This method offers rapid, non-destructive, and efficient advantages, making it suitable for beer quality control and market regulation.

## 1. Introduction

Beer is the world’s most widely consumed alcoholic beverage. Traditional brewing practices follow Germany’s 1516 Beer Purity Law, which limits ingredients to malt, water, hops, and yeast [1,2]. Currently, countries have established clear regulations for key beer metrics such as alcohol content and original wort concentration. Craft beer is typically brewed using all-malt fermentation, emphasizing traditional production process [3,4]. Its extended production cycle ensures exceptional quality. Industrial beer, in contrast, often substitutes malt with low-cost crops like rice or corn and incorporates additives to reduce costs, enabling large-scale standardized production [5,6]. In stark contrast, non-fermented beer is typically produced through non-fermentation blending processes by directly adding artificial ingredients such as edible alcohol, malt powder, and flavorings [7]. Such beers not only lack guaranteed quality but may also pose health risks. Simultaneously, illegal workshops bottle low-grade beer in renowned brand bottles to counterfeit genuine products for exorbitant profits, severely disrupting market order. Therefore, there is an urgent need to develop rapid detection technologies of key indicators and authenticity verification methods for beer to safeguard consumer rights and ensure the healthy development of the industry.

Traditional beer testing methods, such as gas chromatography-mass spectrometry (GC–MS) and isotope ratio mass spectrometry, offer high accuracy in compound detection and authenticity verification [8,9]. Near-infrared spectroscopy (NIRS) technology offers significant advantages through its rapid, non-destructive, and simultaneous multi-component analysis capabilities, substantially reducing costs and labor dependency. Consequently, this method is more suitable than traditional approaches for in situ detection at production sites and rapid applications in market regulation [10]. NIRS analysis faces challenges such as overlapping wavelength variables and the dimensionality disaster, which severely impact the accuracy and performance of quantitative and classification models. To address these issues, dimensionality reduction is typically applied to raw spectral data to eliminate redundant wavelength variables that are uncorrelated or collinear [11,12].

Competitive adaptive reweighted sampling (CARS) and the successive projections algorithm (SPA) are two widely used feature wavelength selection methods in NIRS analysis. They enhance model performance through dynamic optimization and structural simplification, respectively. The CARS algorithm’s core principle emulates the evolutionary concept of “survival of the fittest”. Through iterative optimization and Monte Carlo sampling, it selects feature wavelengths from a large spectral dataset that best predict the target variable, effectively reducing data redundancy [13]. The SPA focuses on resolving multicollinearity among wavelengths. Through a series of orthogonal projection operations, it progressively identifies variables with the lowest linear correlation to the selected wavelength set from unselected wavelengths. This process selects a feature wavelength subset rich in information and low in redundancy, thereby enhancing model accuracy and computational efficiency [14].

With the widespread application of deep learning in spectral analysis, neural networks have demonstrated superior performance over traditional methods in spectral feature transformation and dimensionality reduction due to their powerful feature extraction capabilities [15]. Convolutional neural networks (CNN) and long short-term memory networks (LSTM) have been extensively applied to spectral feature extraction, enabling the construction of high-performance models for spectral qualitative discrimination and quantitative regression [16,17]. However, CNN primarily extracts local features via convolution kernels, showing limited ability to capture long-term dependencies that may exist in spectral data. Conversely, LSTM, with its unique sequence modeling ability, can learn long-term dependencies between spectral bands and capture global spectral features. Yet, they struggle with local feature extraction between bands, making it difficult to leverage the spatial characteristics of the spectrum fully [18]. To fully leverage the strengths of CNN in local feature extraction and LSTM in capturing global spectral characteristics, while effectively addressing the limitations of both architectures in spectral feature extraction, it is crucial to develop a deep feature extraction framework that integrates CNN, LSTM, and the multi-head self-attention mechanism. This approach enables more comprehensive and balanced spectral feature extraction [19].

Additionally, partial least squares regression (PLSR) and partial least squares discriminant analysis (PLS-DA), as classical linear modeling techniques, possess advantages in handling high-dimensional collinear data and are widely applied in the field of spectral modeling [20,21]. However, these linear methods often struggle to capture non-linear features in the data, leading to limited predictive accuracy in complex sample analysis. Consequently, experts have further explored the potential of non-linear supervised learning methods such as support vector machines (SVM) and extreme learning machines (ELM) in spectral modeling. SVM effectively handles non-linear relationships through kernel functions, while ELM demonstrates advantages in rapid calibration and generalization capabilities [22,23].

Based on the above analysis, to meet the practical demands for detecting key indicators and authenticating authenticity of beer, the following research objectives are proposed: (1) Develop a deep feature extraction method integrating CNN-LSTM multi-model fusion, compare it with various traditional feature extraction methods and single neural network feature extraction methods, and establish an efficient spectral feature extraction framework; (2) Apply spectral features extracted via different data dimensionality reduction methods to construct PLSR, SVM, and ELM regression models, analyze their performance in predicting key beer indicator concentrations; (3) Utilize PLS-DA for classification modeling of extracted features, systematically compare the applicability of different dimensionality reduction methods in beer authenticity authentication.

## 2. Materials and Methods

### 2.1. Beer Sample Preparation

The craft and industrial beer samples used in the experiment were sourced from local suppliers. The additives required for the non-fermented beer samples were uniformly purchased from a local food additive store. A total of 138 craft beer samples were collected, representing 23 distinct beer styles from multiple brands. A total of 78 industrial beer samples were collected, representing 13 distinct beer styles from multiple brands. For craft beer and industrial beer, each style comprised two production batches, with three bottles per batch. The production dates of the purchased beer samples were all within two months of the sample collection date. Both craft and industrial beers utilized declared values for alcohol content and original wort concentration as physicochemical indicators; specific declared values are detailed in Table S1. Non-fermented beer samples were prepared using edible alcohol (Liaobe Chemical Plant, Shenyang, China) at 95% vol, maltose (Shandong Tianjiu Biotechnology Co., Ltd., Heze, China) at 99% purity, food flavorings (Guangzhou Zhenweijiang Biotechnology Co., Ltd., Guangzhou, China), anhydrous citric acid (Shandong Yingxuan Industrial Co., Ltd., Weifang, China), and sodium bicarbonate (Langfang Huachen Chemical Co., Ltd., Langfang, China). Distilled water (National Engineering Technology Center for Miscellaneous Grains) was used to dilute these ingredients into four alcohol content levels: 3% vol, 4% vol, 5% vol, and 7% vol. Each alcohol concentration was formulated into five original wort concentrations: 8 °P, 9 °P, 10 °P, 11 °P, and 12 °P. The ratios of other ingredients remained consistent. Each sample was replicated six times, resulting in 120 non-fermented beer samples. Specific formulation ratios are detailed in Table S2. To prevent bubbles from affecting spectral data acquisition during scanning, all beer samples were thoroughly stirred to expel carbon dioxide. They were then stored in sealed reagent bottles in a cool, dry location.

### 2.2. Spectral Data Acquisition

**Figure 1:**
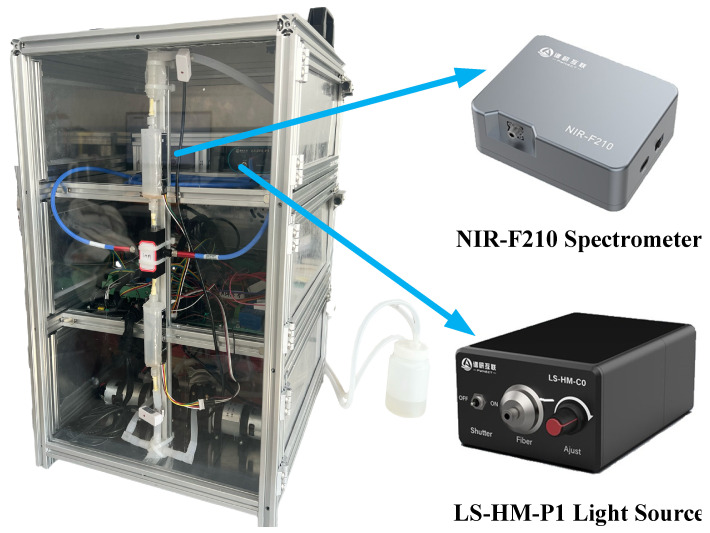
Online liquid-phase near-infrared spectroscopy acquisition system.

This study employed an in-house developed online liquid-phase NIRS data acquisition system to collect spectral data from beer samples. As shown in Figure 1, the system incorporates the NIR-F210 micro-fiber spectrometer (PYNECT, Shenzhen, China) based on digital light processing technology. The instrument was preheated for 30 min before performing transmission-mode spectral scans on beer samples. Air was used as the background, scanned once per hour. The spectral range was 900–1700 nm, with 228 spectral wavelength variables. Each sample underwent one measurement, with each measurement comprising three consecutive scans. The average of these three scans served as the raw spectral data for that sample, yielding a total of 336 raw spectral data points.

### 2.3. Feature Extraction Methods

#### 2.3.1. CARS

CARS simulates the principle of “survival of the fittest” in evolutionary processes to screen out wavelengths most critical for predicting target variables from high-dimensional spectral data [24]. For quantitative regression tasks, CARS combines Monte Carlo sampling with PLSR models. Through adaptive reweighting and competitive selection, it identifies wavelength variables corresponding to the minimum root mean square error of cross-validation (RMSECV), progressively eliminating irrelevant or noisy wavelengths while retaining key variables. For classification tasks, CARS utilizes PLS-DA to select wavelength variable combinations corresponding to the maximum cross-validation accuracy (ACCCV) as the chosen feature wavelengths. This approach reduces model complexity while enhancing model accuracy and robustness.

#### 2.3.2. SPA

SPA filters wavelength variables with high information content and low collinearity from high-dimensional spectral data through iterative projection [25]. SPA reduces collinearity among wavelengths through orthogonal projection and generates candidate wavelength sets. Multiple Linear Regression (MLR) calculates the Predicted Residual Error Sum of Squares (PRESS) for each candidate set via cross-validation, selecting the wavelength subset with the smallest PRESS. Finally, based on variable importance ranking and statistical tests, unimportant wavelengths are sequentially eliminated to determine the minimal yet highly predictive wavelength set.

#### 2.3.3. CNN

CNN utilizes convolutional kernels to capture local spectral features, employs pooling layers to reduce data dimensions for improving computational efficiency and preventing overfitting, and ultimately learns key patterns and features from high-dimensional spectral data through fully connected layers for classification or regression tasks. Compared to traditional methods, it extracts more complex high-level features, enhancing accuracy and generalization capabilities in classification and regression tasks. Optimal configuration of neural network hyperparameters is crucial for achieving peak model performance [26]. The CNN hyperparameters were iteratively optimized for 200 iterations on the full-spectrum data from the calibration set using a Bayesian optimization algorithm (BOA) to enhance model performance. The parameters that were optimized and their search ranges are detailed in Table S3. The objective function for regression feature extraction was the calibration set RMSECV, while the calibration set ACCCV served as the objective function for classification feature extraction. The BOA iteratively optimized CNN parameters for 200 times. The CNN parameters obtained through BOA-optimized feature extraction were then used to construct subsequent quantitative prediction and classification models.

#### 2.3.4. LSTM

LSTM effectively enhances the analytical accuracy of classification or regression tasks by capturing long-term dependencies between wavelengths and extracting complex features related to sequential patterns and dynamic changes in spectral data. Its long-term memory capability enables it to recognize continuous trends across bands, such as variations in the shape and intensity of absorption peaks, thereby strengthening feature extraction from NIRS data [27]. The LSTM’s gating mechanism comprises an input gate, a forget gate, an output gate, and a memory cell, which updates based on current inputs and the hidden state and cell state from the previous time step. Sigmoid activation function maps gate values to the [0, 1] range, while the tanh activation function transforms input data into the [−1, 1] range. Spectral features are ultimately integrated through a fully connected layer. Employing the same objective function for regression and classification feature extraction as used for the CNN, the LSTM hyperparameters were iteratively optimized 200 times using BOA on the full-spectrum data from the calibration set. The optimized parameters and their search ranges are detailed in Table S4. The LSTM parameters obtained through BOA-optimized feature extraction are then utilized for spectral data feature extraction and subsequent modeling.

#### 2.3.5. CNN-LSTM

**Figure 2:**
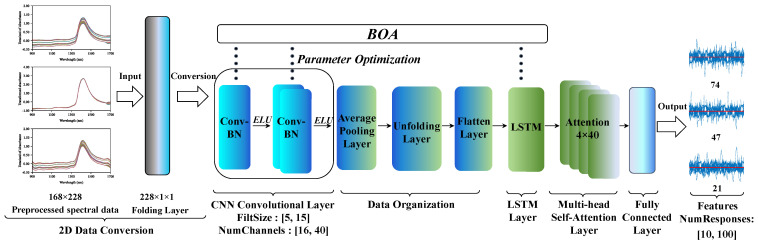
CNN-LSTM Network Structure.

The CNN-LSTM network architecture is illustrated in Figure 2. This architecture begins with a sequence input layer that receives spectral sequence data with dimensions 228 × 1 × 1. Subsequently, a sequence folding layer transforms the data into a format suitable for two-dimensional convolution processing. The CNN feature extraction section comprises two convolutional modules, both of which have kernel sizes and channel counts synchronously optimized via BOA: the first module extracts low-level local features, while the second module doubles the channel count to enhance key feature extraction capabilities. Both modules employ He normal initialization and same padding strategy, where zero padding at boundaries maintains output dimensions consistent with input to preserve spatial information [28]. Subsequently, an exponential linear unit (ELU) activation function is applied to mitigate gradient vanishing while preserving negative values [29]. An average pooling layer performs down-sampling to reduce feature dimensions while retaining primary spectral modes. A sequence unfolding layer reorganizes the convolutional output into a sequence format, followed by a flattening layer that converts the multidimensional feature map into a one-dimensional sequence vector. This ensures the spatially extracted features from the CNN are adapted to the sequential input format of the LSTM, which then receives the input. The number of hidden units in the LSTM layer is also synchronously optimized via BOA to capture long-term dependencies in spectral data and model long-range correlations between wavelengths. Additionally, the multi-head self-attention layer is configured with 4 attention heads and a 40-dimensional key-value space, enhancing focus on key features by computing attention weights. Finally, a fully connected layer integrates global features, outputting a feature vector corresponding to the number of features optimized through 200 iterations of BOA.

The CNN-LSTM hyperparameters were iteratively optimized 200 times using BOA with full spectral data from the calibration set. Specific optimization parameters and their ranges are detailed in Table S5. The RMSECV on the calibration set was used as the objective function for regression modeling feature extraction, and the ACCCV on the calibration set was used as the objective function for classification modeling feature extraction. BOA optimization was employed to obtain CNN-LSTM parameters for spectral data feature extraction.

### 2.4. Modeling Methods and Evaluation Metrics

PLSR excels in regression tasks by fitting high-dimensional, collinear data through latent variables, offering both computational efficiency and strong interpretability, though its non-linear modeling capability is relatively weak. In classification tasks, PLS-DA retains the advantages of PLSR while producing outputs in a discrete format. SVM leverage kernel functions to handle non-linear relationships in both regression and classification tasks. By optimizing parameters through grid search to achieve optimal performance, they ensure high accuracy and robustness, making them suitable for high-precision modeling. ELM employs random weight initialization for both regression and classification tasks. This randomness may compromise stability. Modeling performance is enhanced by grid search adjustments to the number of hidden layer neurons, weight ranges, and bias ranges, making it suitable for rapid modeling and pattern recognition.

The performance of regression models is evaluated using the coefficients of determination ($R^{2}$), root mean square error (RMSE), relative root mean square error (rRMSE) from the calibration set, validation set, and independent test set, along with the residual predictive deviation (RPD) from the validation set and independent test set. When the model exhibits $R^{2}$ > 0.9, rRMSE < 5%, and RPD > 3.0, it indicates excellent prediction accuracy [30]. The performance of classification models is evaluated using the accuracy rate (ACC), precision, recall rate (Recall), macro-averaged F1 score (MF1), and weighted-averaged F1 score (WF1) across the calibration, validation, and independent test sets [31]. Higher values across these five classification metrics indicate greater classification precision.

## 3. Results and Discussion

### 3.1. Data Analysis

NIRS data simultaneously contain absorption information reflecting the chemical composition of the sample and noise signals unrelated to chemical composition [32]. The former represents the chemical properties of the sample, while the latter originates from variations in environmental conditions and the interaction between incident light and the complex physical structure of the sample. Through preprocessing, interference factors such as baseline drift, random noise, and spectral scattering can be effectively removed, thereby enhancing the predictive accuracy and stability of the spectral model. This study employed Savitzky–Golay smoothing (SG), multivariate scattering correction (MSC), standard normal variate (SNV), wavelet denoising (WD), Fourier transform denoising (FTD), and their combinations for spectral preprocessing of beer samples. Performance evaluation of different regression modeling preprocessing methods was conducted using RMSE and RPD based on 10-fold cross-validated PLSR models. Following the same procedure, the optimal preprocessing method for different classification models was determined using ACCCV, precision of cross-validation (PrecisionCV), and recall of cross-validation (RecallCV) based on 10-fold cross-validated PLS-DA models.

**Figure 3:**
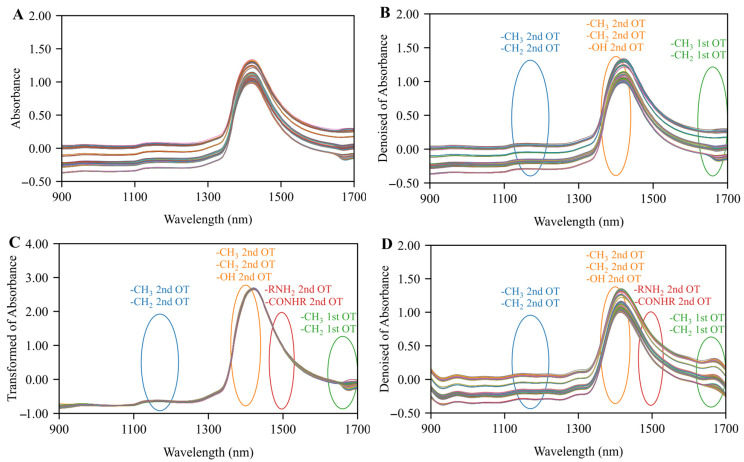
Raw (**A**) and preprocessed spectral data by WT (**B**), SNV (**C**) and the combination of WD and FT (**D**).

As shown in Figure 3A, the raw spectral data exhibited issues such as random noise and baseline drift. Comparative calculations confirmed WD as the optimal preprocessing method for alcohol content indicators in beer sample spectra, with specific performance metrics detailed in Table S6. As shown in Figure 3B, the WD-preprocessed spectral data effectively reduced noise. The 1120–1220 nm band corresponds to the second harmonic of the -CH_3_ and -CH_2_ groups in ethanol within the beer samples, while the 1360–1440 nm band corresponds to the second harmonic of the -CH_3_, -CH_2_, and -OH groups in the beer sample, as well as the 1620–1700 nm band corresponds to the first harmonic of the -CH_3_ and -CH_2_ groups in the beer sample [10]. The optimal pretreatment method for original wort concentration indicators is SNV, with specific performance metrics detailed in Table S7. As shown in Figure 3C, the SNV-pretreated spectral data exhibit a smooth and baseline-leveled morphology. The 1120–1220 nm band corresponds to the second harmonic of the -CH_3_ and -CH_2_ groups of various sugars in the beer sample, while the 1360–1440 nm band corresponds to the second harmonic of -CH_3_, -CH_2_, and -OH groups from various sugars and organic acids in the beer sample. The 1463–1530 nm band corresponds to the second harmonic of the -RNH_2_ groups of amino acids and the -CONHR groups of proteins in the beer sample. The 1620–1700 nm band corresponds to the first harmonic of the -CH_3_ and -CH_2_ groups of various sugars in the beer sample [33]. The optimal preprocessing method for classifying and identifying beer sample spectral data is the combination of WD and FT, with specific performance metrics detailed in Table S8. As shown in Figure 3D, the spectral curves processed by the combination of WD and FT effectively eliminate high-frequency random noise, significantly improving the signal-to-noise ratio and analytical accuracy of the data. Particularly in the 1650–1700 nm band, the preprocessing method of the combination of WD and FT corrected systematic deviations caused by instrument conditions and environmental factors, ensuring the consistency and reliability of the spectral data.

When sample size is limited, effective sample partitioning methods can be employed to select highly representative samples for constructing the calibration set and establishing spectral quantification and classification models [34]. For regression and classification model development, the dataset is divided into a calibration set, validation set, and independent test set at a ratio of 2:1:1. First, the random selection method is employed to randomly select 84 samples from the preprocessed spectral dataset as an external independent test set, used to evaluate the applicability of regression and classification models to unknown samples. The remaining data were then partitioned using the sample set partitioning based on joint X-Y distances (SPXY) method into a calibration set of 168 samples and a validation set of 84 samples. The statistical results of sample partitioning for alcohol content and original wort concentration are shown in Table 1. The coefficient of variation (CV) was used to evaluate the consistency and representativeness of the distribution in the partitioned subsets. The CV values for both measurement indicators in the beer sample calibration set, validation set, and independent test set ranged from 22.93% to 39.42%, indicating suitability for constructing high-performance regression models [35].

### 3.2. Spectral Feature Extraction

#### 3.2.1. CARS Feature Variable Selection

**Figure 4:**
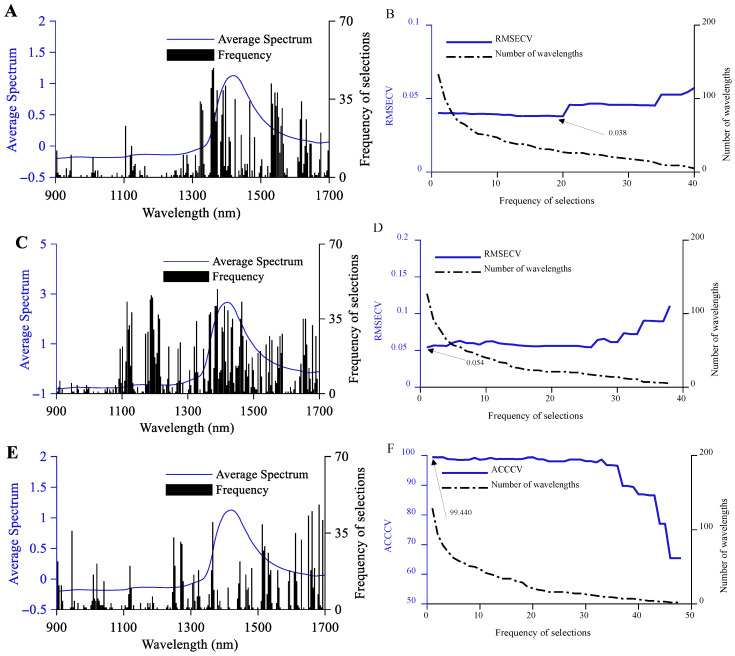
Relationship between the number of variables and RMSECV, ACCCV for CARS Wavelength Selection. (**A**,**C**,**E**) represent the frequency distribution of CARS optimal wavelengths for alcohol content, original wort concentration, and classification identification, respectively. (**B**,**D**) show the relationship between the number of optimal wavelengths for alcohol content and original wort concentration, and the relationship between RMSECV and the number of repeated selections. (**F**) illustrates the relationship between the number of optimal wavelengths for classification detection and the number of repeated selections, and the relationship between ACCCV and the number of repeated selections.

To minimize the impact of randomness inherent in the CARS algorithm on the final modeling results, 50 iterations of the CARS algorithm were performed for preliminary wavelength selection with the full spectral data from the calibration set as input [36]. This process generated preliminary sets of feature wavelengths for both quantitative detection of alcohol and original wort in beer samples, as well as for classification and discrimination. Within these preliminary sets, wavelength variables with higher selection frequencies demonstrated stronger correlation with the detection targets. The preliminary feature wavelength set for alcohol quantification comprised 133 variables, with the most frequently selected wavelength being 1395.51 nm. This corresponds to the second harmonic of the -CH_3_, -CH_2_, and -OH groups in ethanol within the beer samples (Figure 4A). The preliminary feature wavelength set for original wort quantitative detection comprises 127 wavelength variables. The most frequently selected wavelength variable is 1513.21 nm, corresponding to the second harmonic of the -RNH_2_ group in amino acids and the -CONHR group in proteins within the beer sample (Figure 4C). The preliminary set of wavelength features for classification identification contained 129 wavelength variables. The most frequently selected wavelength variable was 1689.42 nm, corresponding to the first harmonic of the -CH_3_ and -CH_2_ groups of ethanol and various sugars in the beer sample (Figure 4E). For both quantitative detection tasks, the relationship between RMSECV and the number of repeated selections was analyzed. As shown in Figure 4B, the RMSECV for the ethanol quantification task first decreased slowly and then increased in a stepwise pattern as the number of selected frequencies increased. At 19 repetitions, the RMSECV of the constructed PLSR model reached its minimum value of 0.038, with 29 selected wavelength variables. As shown in Figure 4D, the RMSECV for the original wort quantitative detection task exhibited a wave-like increase followed by a gradual decrease and finally a sawtooth-like increase as the number of repetitions decreased. When the number of repeated selections was 1, the RMSECV of the constructed PLSR model reached its minimum value of 0.054, with 127 wavelength variables selected. The relationship between ACCCV and the number of wavelength variables for the classification task was established. As shown in Figure 4F, the ACCCV for the classification task decreases in a wave-like pattern followed by a rapid stepwise decline as the number of selected frequencies decreases. When the number of repeated selections is 1, the ACCCV of the constructed PLS-DA model reaches its maximum value of 99.44%, with 129 wavelength variables selected at this point.

#### 3.2.2. SPA Feature Variable Selection

**Figure 5:**
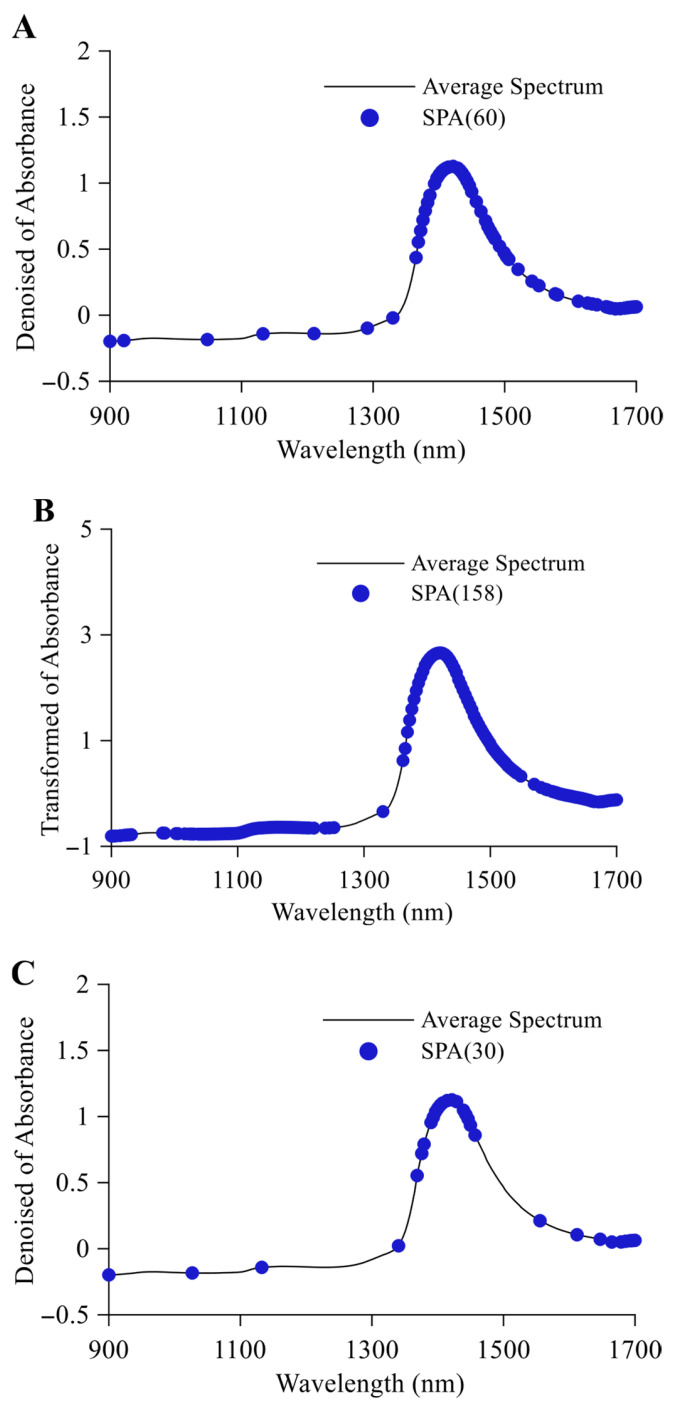
SPA wavelength selection results. (**A**) represents alcohol content, (**B**) denotes original wort concentration, and (**C**) indicates classification discrimination.

SPA exhibits high stability in the selection of optimal spectral feature wavelengths. Using the entire range of wavelengths as the selection scope, a single-run SPA was executed on the full-spectrum data from the beer sample calibration set. This process ultimately identified the optimal wavelength combinations for quantitative alcohol detection, quantitative original wort detection, and classification discrimination in beer samples. As shown in Figure 5, SPA selected 60, 158, and 30 feature wavelengths for alcohol, original wort, and classification discrimination, respectively. The alcohol feature wavelengths are primarily distributed between 1350 and 1700 nm, with some corresponding to the second overtone of the -CH_3_, -CH_2_, and -OH groups and the first overtone of the -CH_3_ and -CH_2_ groups in ethanol within the beer samples. The original wort feature wavelengths exhibit a broader and denser distribution, corresponding to the second overtone of the -CH_3_, -CH_2_, -OH, -RNH_2_, and -CONHR groups, as well as the first overtone of the -CH_3_ and -CH_2_ groups in proteins, amino acids, various organic acids, and multiple sugars within the beer samples. The classification discrimination feature wavelengths are sparsely distributed between 1360 and 1440 nm and between 1620 and 1700 nm, corresponding to the second overtone of the -CH_3_, -CH_2_, and -OH groups and the first overtone of the -CH_3_ and -CH_2_ groups in ethanol, various sugars, and multiple organic acids within the beer samples.

#### 3.2.3. CNN Spectral Feature Extraction

The CNN was trained on the full-spectrum data from the beer sample calibration set, with a maximum of 400 epochs, learning rate reduction every 150 epochs, kernel size of 10, and 32 channels. Other hyperparameters are provided in Table 2. In NIRS analysis, the prediction of alcohol content and original wort concentration, as well as the classification and identification tasks, rely on feature extraction from different functional groups of each indicator. Alcohol focuses on the -CH_3_, -CH_2_, and -OH groups of ethanol, with its clear composition resulting in strong and linearly correlated ethanol feature signals in the spectral data [37]. The BOA-optimized CNN efficiently maps features with 36 fully connected layer responses and a low regularization coefficient. Original wort involves multiple components such as polysaccharides, amino acids, and proteins, with various functional groups, potentially causing overlapping feature signals in the spectral data, which increases the difficulty of feature extraction [33]. The CNN network was optimized with 50 fully connected layer responses and a high learning rate to enhance expressive power. Beer classification and identification require integrating full-spectrum fingerprints, as different beer categories have distinct compositional differences. The CNN network, with 27 fully connected layer responses and a high regularization coefficient, improves generalization ability and effectively suppresses noise.

**Figure 6:**
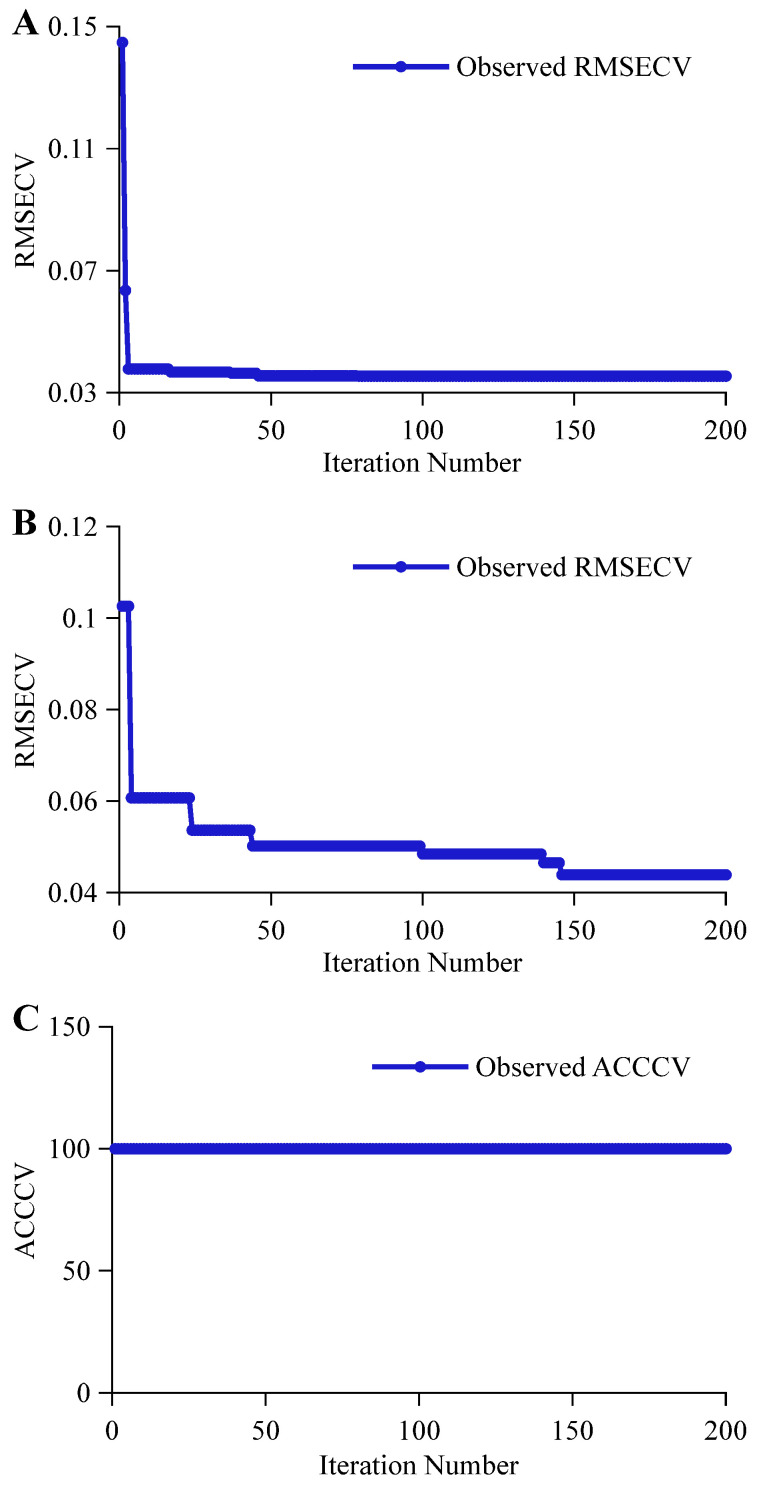
BOA Optimization of the CNN Iteration Process. (**A**,**B**) represent the iterative processes for predicting alcohol content and original wort concentration, respectively, while (**C**) represents the iterative process for the classification discrimination task.

The BOA optimization process for CNN network parameters is illustrated in Figure 6. As shown in Figure 6A, the alcohol content prediction task involves well-defined functional groups with distinct features that exhibit linear correlation with spectral data. Consequently, hyperparameter search proves relatively straightforward, with the iteration curve converging rapidly. The RMSECV achieves its optimal value of 0.0354 at the 46th iteration. As shown in Figure 6B, the original wort concentration prediction task involves numerous components with potentially overlapping functional group combinations, resulting in a more complex search space. The iteration curve shows a trend of gradual optimization, with the RMSECV decreasing from 0.1026 in the first iteration to 0.0607 in the fourth iteration, and progressively optimizing to the best value of 0.0439 at the 24th, 44th, 107th, and 148th iterations. As shown in Figure 6C, the beer classification task exhibits significant compositional differences among beers, resulting in a highly separable search space. The iteration curve follows a linear trend, with the ACCCV reaching 100% at the first iteration and maintaining this value until the 200th iteration.

#### 3.2.4. LSTM Spectral Feature Extraction

The LSTM was trained on the full-spectrum data from the beer sample calibration set, with a maximum of 400 epochs, learning rate reduction every 150 epochs. Other hyperparameters are provided in Table 3. LSTM processes spectral data as sequences, handling spectral data sequentially by wavelength and dynamically extracting sequence features. It progressively constructs features related to spectral patterns, making it suitable for analyzing long-term dependencies and overall trends within spectral data. LSTM relies on global information to capture sequence patterns but exhibits weaker ability to extract local features. Consequently, BOA requires a higher number of fully connected layer responses during hyperparameter optimization for quantitative prediction tasks to integrate dispersed functional group signals and compensate for its limited local feature capture capability [38]. In contrast, the distinct composition and functional group differences in classification tasks may mitigate LSTM’s shortcomings in local feature extraction, enabling it to achieve a lower number of fully connected layer responses compared to CNN.

**Figure 7:**
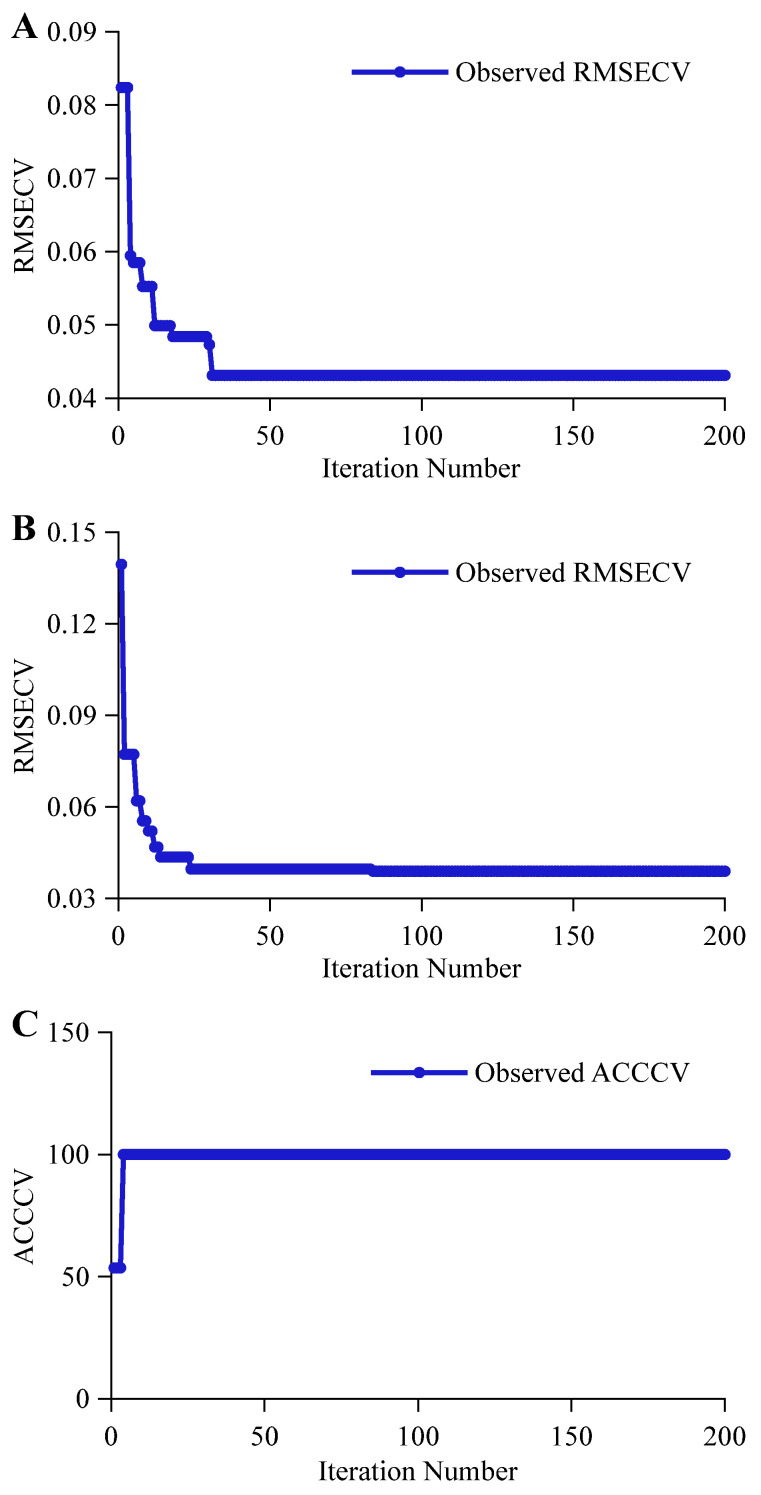
BOA Optimization of the LSTM Iteration Process. (**A**,**B**) represent the iterative processes for predicting alcohol content and original wort concentration, respectively, while (**C**) represents the iterative process for the classification discrimination task.

The iterative process for the alcohol content prediction task is shown in Figure 7A. The RMSECV gradually decreased from 0.0823 in the first iteration, reaching an optimal value of 0.0431 by the 31st iteration. This optimal value remained unchanged throughout the subsequent 170 iterations. The rapid convergence pattern reflects the strong specificity and linear correlation of ethanol’s functional group signals. In contrast, original wort involves complex combinations of multiple functional groups from diverse components such as polysaccharides, proteins, and amino acids. The intricate signal interactions across spectral bands render the BOA search space more multi-modal and challenging. As shown in Figure 7B, compared to the alcohol content prediction task, the original wort concentration prediction task exhibited a more gradual optimization process. Starting from an RMSECV of 0.1400 at the first iteration, it progressively decreased, reaching an optimal value of 0.0389 at the 86th iteration. This optimal value remained stable and unchanged until the 200th iteration. The parameter optimization process for the classification task is shown in Figure 7C. Although the significant differences among functional groups may mitigate the LSTM’s weakness in local feature extraction, it still requires more iterations than CNN to adapt to the distinct yet dispersed functional group fingerprints. Compared to CNN achieving 100% ACCCV in the first iteration, LSTM needed four iterations to optimize ACCCV to 100%.

#### 3.2.5. CNN-LSTM Spectral Feature Extraction

The CNN-LSTM was trained on the full-spectrum data from the beer sample calibration set. The BOA optimized the CNN-LSTM hyperparameters, with results detailed in Table 4. The CNN-LSTM combines CNN’s local feature extraction, LSTM’s long-term dependency capture, and the multi-head attention mechanism to enhance feature extraction efficiency. In the alcohol content prediction task, the fusion architecture integrates CNN local features, LSTM long-term dependency capture, and the dimensions introduced by multi-head attention mechanisms, ensuring the capture of ethanol group features. However, multi-layer interactions increase network complexity, requiring more fully connected layer response units to process the integrated high-dimensional features. Consequently, the number of fully connected layer responses in the CNN-LSTM network (74) exceeds those in the CNN (36) and LSTM (50) networks [39]. When optimizing the CNN-LSTM network with BOA, the convolutional kernel for the alcohol content metric was set to 7, while the original wort concentration metric used a 10 kernels. The larger number of kernels for original wort concentration prediction task enables effective resolution of potentially overlapping group signals from multiple components. This expanded receptive field covers a broader wavelength range, enhancing feature extraction robustness [40]. The BOA-optimized CNN-LSTM network reduces the number of fully connected layer responses to 47 by employing larger convolutional kernels alongside low channel counts and low regularization. This makes the model more efficient when processing high-dimensional data with potentially overlapping functional group signals. Classification tasks rely on full spectral fingerprints with distinct functional group signals. The optimized CNN-LSTM focuses on discriminating boundaries, resulting in fully connected layer responses comparable to those of standalone CNN and LSTM. The synergistic integration of CNN, LSTM, and attention mechanisms enhances global feature extraction capability and efficiency, enabling adaptation to functional group characteristics across diverse tasks. Comparing parameters of BOA-optimized CNN, LSTM, and CNN-LSTM reveals that BOA yields distinctly optimized results for balancing functional group properties with architectural efficiency when applied to deal with different modeling tasks. This phenomenon underscores the necessity of neural network hyperparameter optimization.

**Figure 8:**
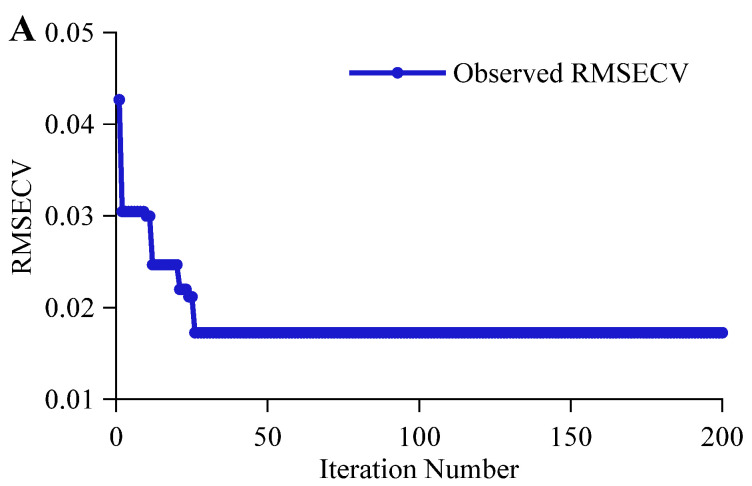

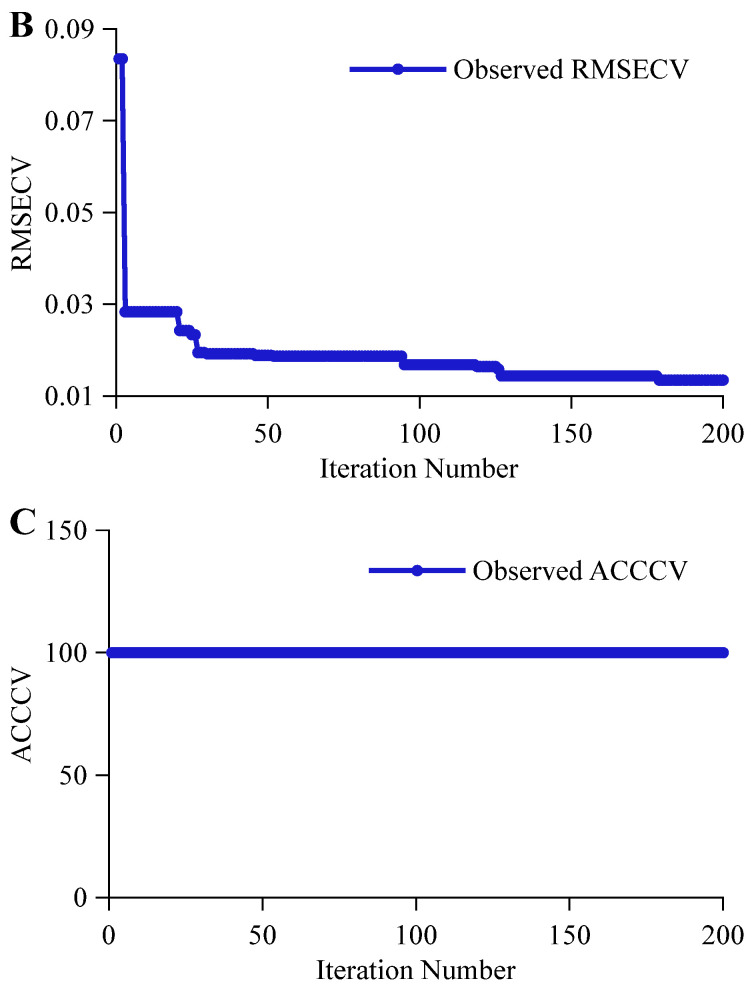
BOA optimization of the CNN-LSTM Iteration Process. (**A**,**B**) represent the iterative processes for predicting alcohol content and original wort concentration, respectively, while (**C**) represents the iterative process for the classification discrimination task.

The iterative process of BOA-optimized CNN-LSTM for alcohol content prediction task is shown in Figure 8A. The RMSECV rapidly decreased from 0.0427 in the first iteration to 0.0305 in the second iteration, reaching an optimal value of 0.0172 in the 26th iteration. The optimal value remained stable thereafter without further updates. The CNN-LSTM achieves rapid convergence in alcohol content prediction task by leveraging CNN’s efficient local feature capture, LSTM’s long-term dependency extraction on local features, and the multi-head attention mechanism’s focus on key functional groups. The optimal objective function value (RMSECV) of the CNN-LSTM network is 0.0172, significantly lower than that of the CNN or LSTM alone. As shown in Figure 8B, the iterative optimization process for original wort concentration prediction task exhibits a stepwise improvement pattern. The RMSECV rapidly decreased from 0.0834 in the first iteration to 0.0283 in the third iteration. Subsequently, the optimal value was updated through multiple iterations, reaching the final optimal RMSECV of 0.0134 at the 191st iteration. The extremely low regularization coefficient obtained through BOA optimization adapts to non-linear data, while the higher learning rate and decay factor support progressive fine-tuning, enhancing the CNN-LSTM’s efficient processing capability for complex functional groups. As shown in Figure 8C, during the BOA-optimized CNN-LSTM for the classification task, the objective function value (ACCCV) reached 100% from the first iteration and remained stable at 100% throughout the subsequent 200 iterations. Although both CNN and CNN-LSTM achieved 100% ACCCV in the first iteration, the CNN-LSTM attained this performance with fewer fully connected layer responses. Due to differences in functional group characteristics and feature extraction difficulty across the three tasks, CNN, LSTM, and CNN-LSTM exhibited varying adaptability, resulting in distinct hyperparameter requirements [41]. BOA effectively optimizes hyperparameters by adapting to task complexity and model architecture, thereby enhancing feature extraction and modeling performance.

### 3.3. Model Construction and Evaluation

#### 3.3.1. Quantitative Forecast Results and Analysis

By establishing PLSR, SVM, and ELM models, we analyzed the modeling performance of alcohol content and original wort concentration using full-spectrum (Full) and spectral features extracted by CARS, SPA, CNN, LSTM, and CNN-LSTM. Performance evaluation was conducted using the $R^{2}$, RMSE, rRMSE, and RPD across different quantitative prediction models for alcohol content and original wort concentration, utilizing calibration, validation, and independent test sets.

As shown in Table 5, in constructing quantitative detection models for beer alcohol content, the wavelength variables optimized by CARS were reduced by 87% compared with the full spectrum. The constructed PLSR and ELM regression models outperformed the full-spectrum modeling approach, while the SVM model demonstrated reduced performance compared to full-spectrum modeling. This may stem from SVM’s high dependency on kernel functions and feature space. While CARS selected variables most relevant to the target variable, it potentially disrupted the overall structure of the original feature space. Consequently, the optimized wavelength subset may prove unsuitable for SVM kernel function mapping [42]. Wavelength variables optimized via SPA were reduced by over 73%, with the constructed PLSR, SVM, and ELM regression models all outperforming full-spectrum modeling. Traditional CNN and LSTM direct regression modeling performed worse than PLSR, SVM, and ELM models built after feature extraction via CNN and LSTM. This is attributed to that CNN and LSTM captured spectral data features by extracting local features and long-term dependencies, respectively. The performance of PLSR, SVM, and ELM regression models constructed this way far exceeded that of regression models built after wavelength selection via traditional CARS and SPA. As a hybrid of both architectures, the CNN-LSTM network efficiently extracts deep features from raw spectral data that simultaneously incorporate local feature information and long-term dependencies. These highly abstracted and integrated features are unattainable by traditional methods [19]. The PLSR, SVM, and ELM models constructed using these extracted deep features outperform regression models built with features extracted by other methods.

**Figure 9:**
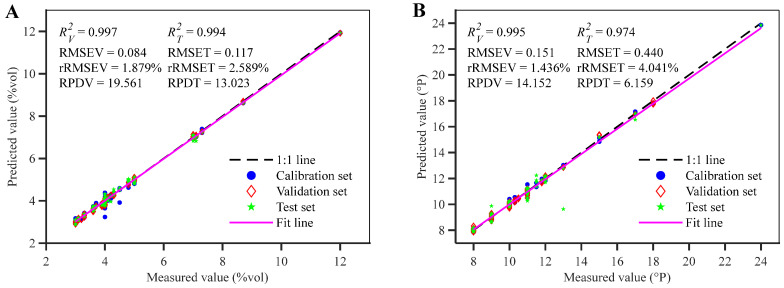
Performance evaluation plot of alcohol content (**A**) and original wort concentration (**B**) models. $R_{v}^{2}$, $R_{t}^{2}$, RMSEV, RMSET, rRMSEV, rRMSET represent $R^{2}$, RMSE and rRMSE of validation and test sets, respectively. RPDV and RPDT represent RPD of validation and test sets.

The optimal regression model for predicting beer alcohol content is an SVM model constructed after deep feature extraction via CNN-LSTM. Its $R^{2}$ for the calibration set, validation set, and independent test set are 0.995, 0.997, and 0.994, respectively, with RMSE values of 0.120, 0.084, and 0.117. The rRMSE was 2.664%, 1.879%, and 2.589%, respectively. The RPD for the validation and independent test sets was 19.561 and 13.023, respectively. The model significantly outperforms multiple regression models based on full-spectrum data and other feature extraction methods. Its high alcohol prediction accuracy arises from the strong, distinctive absorption bands of ethanol (-CH_3_, -CH_2_, and -OH groups) in the near-infrared spectrum, which show minimal overlap with other beer components in the 900–1700 nm range. This enables the CNN-LSTM to easily extract ethanol features, ensuring the regression model meets the need for rapid alcohol detection in beer. [37]. The diagonal distribution plot of actual versus predicted values for the CNN-LSTM-SVM model shows that, except for a few calibration set data points, the calibration set, validation set, and independent test set data points for beer alcohol content are largely located along the 1:1 line (Figure 9A). Furthermore, the fitted line closely aligns with the 1:1 line. For unknown samples in the validation and independent test sets, the model demonstrates good stability and robustness, further indicating that the CNN-LSTM network can effectively extract deep features from NIRS data. The combination of CNN-LSTM network with a suitable regression model can achieve high predictive performance. As shown in the distribution plot of actual versus predicted values in Figure 9A, the independent test set constructed using the SPXY method exhibits a wide distribution with pronounced random characteristics. The CNN-LSTM-SVM model achieves an $R^{2}$ exceeding 0.99, an rRMSE below 2.59%, and an RPD greater than 13 for the independent test set. This indicates the model possesses strong capability for measuring unknown samples and high robustness. A *t*-test performed using the Matlab ttest function on the actual and predicted beer alcohol content values returned a result of 0, with a significance level probability of 0.317. This is less than the critical value *P*_0.05_(335) = 1.967, indicating no significant difference between the actual and predicted alcohol content values and good consistency [35].

**Figure 10:**
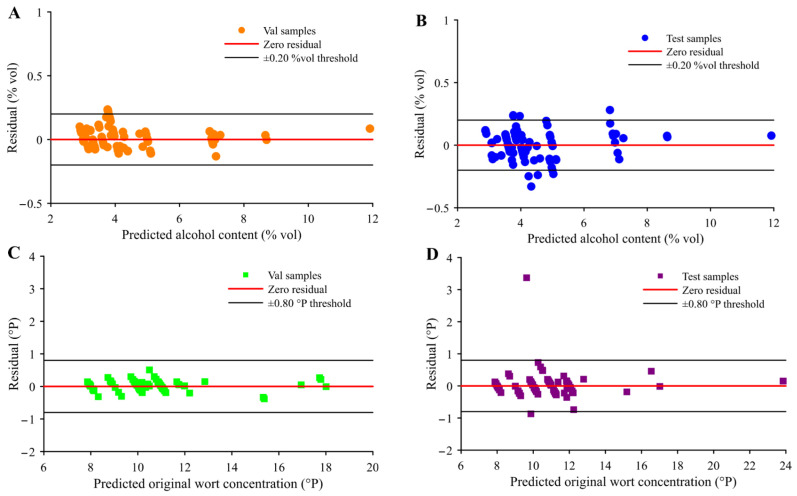
Confidence Interval Plots for Alcohol Content (**A**,**B**) and Original Wort Concentration (**C**,**D**) Models.

To further assess the model’s reliability and robustness, residual analysis and uncertainty quantification were performed. Confidence intervals for the validation and independent test sets are shown in Figure 10A and Figure 10B, respectively. Validation set residuals ranged from −0.132% vol to 0.236% vol, while independent test set residuals spanned −0.330% vol to 0.281% vol. Notably, 96.4% of validation samples had absolute residuals ≤ 0.20% vol, versus 89.3% in the test set. These findings confirm that the CNN-LSTM-SVM model delivers high accuracy, consistency, and predictive robustness in alcohol content estimation. In summary, the CNN-LSTM network constructed by integrating CNN and LSTM represents an efficient and reliable spectral feature extraction method. The SVM quantitative calibration model developed by combining NIRS with CNN-LSTM can be effectively applied for the rapid detection of beer alcohol content [10].

As shown in Table 6, when constructing a quantitative detection model for beer original wort concentration, the regression model built after wavelength variable selection via CARS exhibited reduced performance compared to the full-spectrum modeling approach. The main reason is that his study did not exclude outlier samples. Thus, individual anomalous samples may have caused errors in the CARS algorithm during variable selection, thereby diminishing the model’s predictive capability and robustness. Similarly, the PLSR model constructed after wavelength variable selection via SPA exhibited reduced performance compared to the full-spectrum model, while the non-linear SVM and ELM models outperformed the full-spectrum model. This stems from SPA potentially disrupting the latent variable structure relied upon by the PLSR model during multicollinearity elimination, resulting in suboptimal PLSR prediction performance [43]. Regarding feature dimensionality reduction for original wort concentration, CNN and LSTM reduced feature dimensions by 58% and 85% compared to the full spectrum, respectively, while traditional CARS and SPA achieved reductions of 44% and 31%. This demonstrates the superior feature dimensionality reduction capability of neural networks [16]. Regression models constructed using CNN and LSTM-extracted features consistently outperformed full-spectrum modeling, demonstrating their stability in feature extraction. However, the SVM models built with these features performed below SVM models constructed using CARS and SPA-selected optimal feature wavelengths. This may stem from the SVM kernel mechanism’s inability to fully adapt to the abstract features extracted by the two standalone neural networks, resulting in inferior generalization performance compared to SVM models built on CARS and SPA-selected feature subsets. Regarding modeling outcomes from different feature extraction methods, CNN-LSTM demonstrated the best overall performance. The regression prediction models constructed using this method outperformed those built with other feature extraction approaches while effectively balancing model performance and data dimensionality reduction [19].

The optimal regression model for predicting beer original wort concentration is an SVM model constructed after deep feature extraction via CNN-LSTM, with $R^{2}$ of 0.998, 0.995, and 0.974, RMSE of 0.135, 0.151, and 0.440, rRMSE of 1.240%, 1.436%, and 4.041% for the calibration, validation, and independent test sets, respectively. The RPDs are 14.152 and 6.159 for validation, and independent test sets, respectively. This model significantly outperforms regression models constructed using full-spectrum data or other feature extraction strategies. Its superior performance is primarily attributed to the CNN-LSTM architecture integrating multi-head self-attention, which effectively resolves heavily overlapping absorption bands of multiple chemical groups (-CH_2_, -CH_3_, -OH, -RNH_2_, -CONHR). This facilitates the extraction of local features and long-range dependencies beyond the reach of conventional wavelength selection methods, while dynamically attending to spectral regions associated with key functional groups. By fusing local and global chemical information, the model achieves representation capability unattainable by traditional approaches, enabling rapid and reliable determination of beer original wort concentration [33]. The diagonal distribution plot of actual versus predicted values from the CNN-LSTM-SVM model shows that most data points for original wort concentration cluster near the 1:1 line (Figure 9B). Samples from the calibration and validation sets align closely with this line, while sample 7 in the independent test set exhibits significant deviation. This deviation may stem from instrument noise during data acquisition and the absence of outlier removal strategies in this study. Additionally, the difference between the fitted line and the 1:1 line is relatively small, indicating stable model fitting performance. The CNN-LSTM-SVM model achieved an $R^{2}$ exceeding 0.97, an rRMSE below 4.05%, and an RPD greater than 6.15 in the independent test set, indicating robust performance in measuring unknown samples. The *t*-test result comparing actual and predicted original wort concentrations returned to a value of 0, with a significance probability of 0.445—below the critical value *P*_0.05_(335) = 1.967. Thus, no significant difference exists between actual and predicted original wort concentrations [35]. Further validation of model uncertainty was performed via residual analysis. Confidence intervals for the validation and independent test sets are presented in Figure 10C and Figure 10D, respectively. Original wort concentration residuals ranged from −0.381 °P to 0.507 °P in the validation set, and from −0.870 °P to 3.369 °P in the independent test set. Notably, all validation samples had absolute residuals ≤ 0.80 °P, with 97.6% of independent test samples also below this threshold. These results confirm that the CNN-LSTM-SVM model delivers excellent predictive performance for original wort concentration, with reasonable residual distribution and high stability. In summary, the CNN-LSTM network effectively extracts deep features for predicting beer original wort concentration. Combined with NIRS and SVM regression prediction models, it demonstrates significant advantages in achieving rapid and reliable detection of beer original wort concentration.

#### 3.3.2. Classification Results and Analysis

By constructing PLS-DA models incorporating various wavelength selection and feature extraction methods, we analyzed the modeling performance of Full, CARS, SPA, CNN, LSTM, and CNN-LSTM. Performance evaluation was conducted using ACC, Precision, Recall, MF1, and WF1 metrics across calibration, validation, and independent test datasets.

As shown in Table 7, the full-spectrum PLS-DA model achieved 100% for multiple evaluation metrics in both the calibration and validation sets. For the independent test set, the ACC was 98.809%, precision was 99.074%, Recall was 98.889%, MF1 was 98.965%, and WF1 was 98.808%. The primary reason linear models achieve high metrics lies in the differences in beer ingredients and production processes [4]. Industrial beers typically incorporate adjuncts like rice and corn with lower malt ratios, resulting in relatively lower protein, amino acid, and complex carbohydrate content, thus presenting a simpler matrix. In contrast, craft beer strictly adheres to all-malt brewing and heavily utilizes hops. Its spectral characteristics reflect richer malt-derived sugars, peptides, and complex flavor compounds like esters and alcohols produced by yeast fermentation, resulting in a complex spectral signal. Non-fermented beer, however, is blended from edible alcohol and additives, resulting in a spectral profile markedly different from that of naturally fermented products.

The CARS algorithm was employed for feature wavelength selection, and a PLS-DA discriminant model was constructed based on the selected feature wavelengths, ultimately reducing the feature dimension to 129 dimensions. Results indicate that this model exhibits classification performance comparable to the full-spectrum PLS-DA model, demonstrating that the feature wavelengths screened by CARS effectively represent most of the discriminative information in the original spectrum. Feature extraction using the SPA significantly reduced the dimension to 30. The constructed PLS-DA model achieved 100% for all five performance evaluation metrics on the calibration set, validation set, and independent test set, demonstrating excellent classification capability and generalization performance. Compared to traditional feature selection methods, neural networks exhibit superior performance in data dimensionality reduction. Specifically, the CNN model compressed features to 27 dimensions, the LSTM model reduced them to 24 dimensions, and the CNN-LSTM model further decreased dimensions to 21. Neural network feature extraction not only achieves more extreme feature compression but also possesses stronger feature abstraction and representation capabilities [44]. Features extracted via neural networks were employed to construct classification models, achieving 100% across all five performance metrics on the calibration set, validation set, and independent test set. This outcome demonstrates that neural network-derived spectral features possess superior discriminative power and robustness, significantly enhancing the classification capabilities of traditional machine learning models. This approach provides a more advanced and flexible technical pathway for processing high-dimensional spectral data [45]. The PLS-DA model built on neural network-extracted features achieved 100% across all five evaluation metrics in the calibration, validation, and independent test sets. This superior efficiency is primarily attributed to the pronounced spectral differences among the three beer categories: craft beer exhibits a complex natural fermentation fingerprint rich in esters, higher alcohols, and peptides; industrial beer displays a simplified spectral profile due to adjunct addition and shorter fermentation cycles; while non-fermented beer, dominated by added ethanol and flavorings, presents a highly uniform matrix. Such marked inter-class separability renders the classification task inherently discriminative, even for linear models. Consequently, neural network-derived features demonstrate superior discriminative power and robustness, significantly enhancing the performance of conventional machine learning classifiers and offering an advanced, flexible pathway for high-dimensional spectral analysis.

### 3.4. Advantages and Limitations

Compared to traditional methods such as GC–MS, the NIRS-based approach demonstrates significant advantages in cost-effectiveness and practicality. Although traditional methods offer high accuracy, they rely on expensive instruments, require complex sample preparation, are time-consuming, and must be operated by trained personnel in a laboratory. In contrast, NIRS is inherently rapid, non-destructive, and requires no complex sample preparation. The self-developed online liquid-phase NIRS system (Figure 1) uses a commercial micro-spectrometer, resulting in low hardware costs. More importantly, once calibrated, subsequent analyses consume negligible chemicals and require minimal manual intervention, significantly reducing per-sample cost and time—highlighting superior economic efficiency and throughput.

The experimental design prioritizes industrial feasibility. First, each sample undergoes a single spectral scan, simulating rapid single-sample analysis in online industrial settings. Second, the system in Figure 1 integrates transmissive detection with inline piping, enabling in situ, real-time monitoring of key indicators on production lines. Its compact design supports seamless integration into existing infrastructure. Thus, this method outperforms traditional laboratory techniques in cost and practicality while offering strong potential for industrial deployment.

Traditional methods like CARS and SPA operate at the wavelength level, eliminating collinearity and noise to select variables with clear physical significance—effective when absorption bands are sparse and linearly correlated with targets. In contrast, deep learning methods (CNN, LSTM, and CNN-LSTM fusion networks) extract high-level abstract features. CNN excels at local pattern capture, LSTM at long-range dependency modeling, performing well with uniform or distinct functional groups. The CNN-LSTM with multi-head attention, however, generates highly compressed yet informative features, preserving local details while fully exploiting inter-wavelength dependencies, enabling effective extraction of complex functional group information.

Despite its promise, several limitations should be noted. First, alcohol content and original wort concentration for craft and industrial beers were derived from manufacturer labels, and non-fermented samples from formulation specifications—potentially introducing approximations versus standard analytical values, affecting calibration accuracy. Future studies should use validated reference methods. Second, the samples primarily come from Chinese suppliers, covering mainstream industrial beers and emerging craft beers, but they fail to reflect global diversity, limiting their international applicability. Third, all data originated from a single experimental setup without independent external validation, risking reduced generalizability across instruments or conditions. Future work should expand sample diversity and prioritize cross-validation with external datasets—essential for robust, real-world spectral models.

## 4. Conclusions

This study successfully established a beer quality analysis method based on NIRS and neural network feature extraction, achieving high-precision quantitative detection of alcohol content and original wort concentration, as well as beer authenticity verification. The CNN-LSTM feature extraction combined with SVM regression modeling demonstrated optimal performance. The alcohol content prediction model achieved an $R^{2}$ exceeding 0.99 across the calibration set, validation set, and external independent test set, with an rRMSE below 2.67%. For the original wort concentration prediction model, the $R^{2}$ exceeded 0.97 across the calibration, validation, and external independent test sets, with an rRMSE below 4.05%. For classification, the PLS-DA model built with deep features extracted via CNN-LSTM achieved 100% accuracy in distinguishing craft, industrial, and non-fermented beers. This approach outperformed traditional wavelength selection methods and single neural network feature extraction in dimensionality reduction and modeling performance. Through effective feature reduction and integrated modeling, this NIRS-based approach overcomes the limitations of traditional detection methods—complex operation, time-consuming processes, and reliance on professional expertise. It demonstrates the superior reliability of combining NIRS with CNN-LSTM feature extraction for detecting key beer quality indicators, providing a rapid, non-destructive, and efficient solution for quality control and market regulation in the beer industry.

## Figures and Tables

**Table 1:** Alcohol Content and Original Gravity Statistics.

Indicator	Subsets	Number	Mean (%vol/°P)	Max (%vol/°P)	Min (%vol/°P)	Standard Deviation (%vol/°P)	Coefficient of Variation (%)
Alcohol content	Calibration set	168	4.497	12	3	1.773	39.426
	Validation set	84	4.495	12	3	1.537	34.193
	Test set	84	4.532	12	3	1.624	35.834
Original wort	Calibration set	168	10.865	24	8	2.705	24.896
	Validation set	84	10.613	24	8	2.434	22.934
	Test set	84	10.879	24	8	2.719	24.993

**Table 2:** CNN Parameter Optimization Results.

Key Indicators	Parameters	Results
Alcohol content	numResponses	36
	InitialLearnRate	0.0011024
	L2Regularization	2.0555 × 10^−10^
	LearnRateDropFactor	0.66659
	Parameter count	263,044
Original wort	numResponses	50
	InitialLearnRate	0.007966
	L2Regularization	6.6968 × 10^−5^
	LearnRateDropFactor	0.86817
	Parameter count	365,202
Classification and Identification	numResponses	27
	InitialLearnRate	0.0017568
	L2Regularization	0.0033696
	LearnRateDropFactor	0.4984
	Parameter count	197,371

numResponses, InitialLearnRate, L2Regularization, and LearnRateDropFactor represent the number of responses in the fully connected layer, the initial learning rate, the L2 regularization factor, and the learning rate drop factor, respectively.

**Table 3:** LSTM Parameter Optimization Results.

Key Indicators	Parameters	Results
Alcohol content	numResponses	50
	InitialLearnRate	0.0050306
	L2Regularization	4.9258 × 10^−7^
	LearnRateDropFactor	0.61321
	Parameter count	58,350
Original wort	numResponses	95
	InitialLearnRate	0.0051512
	L2Regularization	3.1168 × 10^−9^
	LearnRateDropFactor	0.89368
	Parameter count	132,240
Classification and Identification	numResponses	26
	InitialLearnRate	0.010598
	L2Regularization	9.7163 × 10^−5^
	LearnRateDropFactor	0.88148
	Parameter count	27,222

numResponses, InitialLearnRate, L2Regularization, and LearnRateDropFactor represent the number of responses in the fully connected layer, the initial learning rate, the L2 regularization factor, and the learning rate drop factor, respectively.

**Table 4:** CNN-LSTM Parameter Optimization Results.

Key Indicators	Parameter	Result
Alcohol content	numResponses	74
	FiltSize	7
	numChannels	37
	MaxEpochs	294
	numHiddenUnits	38
	InitialLearnRate	0.0041
	LearnRateDropPeriod	114
	L2Regularization	9.8567 × 10^−6^
	LearnRateDropFactor	0.8671
	Parameter count	111,933
Original wort	numResponses	47
	FiltSize	10
	numChannels	18
	MaxEpochs	100
	numHiddenUnits	32
	InitialLearnRate	0.0051
	LearnRateDropPeriod	86
	L2Regularization	1.6383 × 10^−8^
	LearnRateDropFactor	0.8783
	Parameter count	47,737
Classification and Identification	numResponses	21
	FiltSize	5
	numChannels	22
	MaxEpochs	125
	numHiddenUnits	32
	InitialLearnRate	0.0033777
	LearnRateDropPeriod	99
	L2Regularization	2.3756 × 10^−8^
	LearnRateDropFactor	0.57755
	Parameter count	84,323

numResponses, FiltSize, numChannels, MaxEpochs, numHiddenUnits, InitialLearnRate, LearnRateDropPeriod, L2Regularization, and LearnRateDropFactor represent the number of responses in the fully connected layer, the size of the convolutional kernel, the number of convolutional channels, the maximum number of iterations, the number of hidden units in the LSTM, the initial learning rate, the learning rate drop period, the L2 regularization factor, and the learning rate drop factor, respectively.

**Table 5:** Evaluation Criteria for Quantitative Models of Alcohol Content.

Model	Method	Dimension	$R_{c}^{2}$	$R_{v}^{2}$	$R_{t}^{2}$	RMSEC	RMSEV	RMSET	rRMSEC	rRMSEV	rRMSET	RPDV	RPDT	LVs
CNN	CNN	36	0.976	0.968	0.956	0.273	0.288	0.320	6.075	6.410	7.060	5.693	4.781	N/A
LSTM	LSTM	50	0.965	0.979	0.955	0.328	0.237	0.325	7.301	5.263	7.179	6.864	4.723	N/A
PLSR	Full	228	0.964	0.950	0.947	0.334	0.361	0.353	7.420	8.039	7.783	4.559	4.337	10
	CARS	29	0.966	0.954	0.952	0.325	0.347	0.335	7.225	7.722	7.399	4.699	4.562	10
	SPA	60	0.967	0.952	0.950	0.323	0.353	0.343	7.176	7.849	7.571	4.656	4.455	10
	CNN	36	0.973	0.964	0.955	0.289	0.304	0.323	6.422	6.767	7.132	5.420	4.736	9
	LSTM	50	0.977	0.965	0.957	0.266	0.304	0.318	5.911	6.753	7.018	5.424	4.829	15
	CNN-LSTM	74	0.988	0.991	0.980	0.194	0.157	0.214	4.314	3.483	4.721	10.532	7.143	14
SVM	Full	228	0.980	0.978	0.971	0.252	0.238	0.260	5.612	5.304	5.742	6.903	5.884	N/A
	CARS	29	0.981	0.975	0.963	0.245	0.257	0.293	5.445	5.725	6.500	6.457	5.252	N/A
	SPA	60	0.989	0.986	0.973	0.185	0.194	0.250	4.121	4.305	5.506	8.745	6.186	N/A
	CNN	36	0.998	0.996	0.991	0.079	0.108	0.145	1.759	2.406	3.192	16.357	10.568	N/A
	LSTM	50	0.994	0.996	0.985	0.136	0.100	0.185	3.017	2.223	4.079	17.266	8.271	N/A
	CNN-LSTM	74	0.995	0.997	0.994	0.120	0.084	0.117	2.664	1.879	2.589	19.561	13.023	N/A
ELM	Full	228	0.977	0.965	0.950	0.269	0.301	0.343	5.991	6.688	7.571	5.478	4.481	N/A
	CARS	29	0.972	0.971	0.962	0.295	0.274	0.299	6.551	6.085	6.601	6.016	5.120	N/A
	SPA	60	0.979	0.969	0.954	0.255	0.283	0.328	5.664	6.305	7.231	5.715	4.702	N/A
	CNN	36	0.993	0.990	0.981	0.150	0.163	0.211	3.329	3.623	4.660	11.253	7.243	N/A
	LSTM	50	0.991	0.989	0.971	0.172	0.173	0.261	3.817	3.841	5.763	9.350	5.973	N/A
	CNN-LSTM	74	0.992	0.994	0.983	0.163	0.130	0.202	3.615	2.893	4.452	12.778	7.574	N/A

$R_{c}^{2}$, $R_{v}^{2}$, $R_{t}^{2}$, RMSEC, RMSEV, RMSET, rRMSEC, rRMSEV, rRMSET represent $R^{2}$, RMSE and rRMSE of calibration, validation and test sets, respectively. RPDV and RPDT represent RPD of validation and test sets. LVs are the principal components extracted from PLSR. The optimal number of LVs is determined by the minimum PRESS value obtained during cross-validation, are not applicable to CNN, LSTM, SVM and ELM models (denoted as N/A).

**Table 6:** Quantitative Model Evaluation Indicators for Original Wort Concentration.

Model	Method	Dimension	$R_{c}^{2}$	$R_{v}^{2}$	$R_{t}^{2}$	RMSEC	RMSEV	RMSET	rRMSEC	rRMSEV	rRMSET	RPDV	RPDT	LVs
CNN	CNN	95	0.958	0.955	0.874	0.555	0.513	0.961	5.107	4.832	8.831	4.720	2.903	N/A
LSTM	LSTM	35	0.950	0.944	0.928	0.628	0.504	0.725	5.762	4.782	6.662	4.379	3.793	N/A
PLSR	Full	228	0.892	0.865	0.852	0.886	0.888	1.039	8.154	8.371	9.552	2.724	2.601	10
	CARS	127	0.899	0.861	0.855	0.856	0.903	1.029	7.880	8.505	9.457	2.681	2.627	10
	SPA	158	0.889	0.860	0.830	0.898	0.906	1.113	8.265	8.532	10.229	2.672	2.429	10
	CNN	95	0.975	0.966	0.942	0.422	0.447	0.648	3.887	4.211	5.959	5.416	4.228	11
	LSTM	35	0.975	0.969	0.945	0.440	0.376	0.632	4.041	3.562	5.806	5.810	4.300	11
	CNN-LSTM	47	0.995	0.989	0.964	0.202	0.219	0.510	1.854	2.074	4.689	9.728	5.316	12
SVM	Full	228	0.997	0.993	0.973	0.137	0.203	0.445	1.264	1.910	4.088	11.985	6.077	N/A
	CARS	127	0.998	0.996	0.967	0.134	0.152	0.493	1.237	1.429	4.530	13.756	5.498	N/A
	SPA	158	0.997	0.994	0.974	0.135	0.181	0.439	1.245	1.701	4.039	13.410	6.167	N/A
	CNN	95	0.997	0.990	0.965	0.140	0.238	0.507	1.268	2.428	4.954	10.192	5.385	N/A
	LSTM	35	0.997	0.992	0.933	0.160	0.185	0.698	1.471	1.758	6.417	11.583	3.878	N/A
	CNN-LSTM	47	0.998	0.995	0.974	0.135	0.151	0.440	1.240	1.436	4.041	14.152	6.159	N/A
ELM	Full	228	0.942	0.904	0.903	0.651	0.750	0.843	5.993	7.070	7.752	3.252	3.272	N/A
	CARS	127	0.935	0.906	0.895	0.689	0.742	0.876	6.346	6.990	8.057	3.265	3.130	N/A
	SPA	158	0.963	0.949	0.914	0.516	0.549	0.791	4.752	5.170	7.276	4.431	3.416	N/A
	CNN	95	0.987	0.955	0.939	0.308	0.512	0.668	2.836	4.825	6.143	4.736	4.068	N/A
	LSTM	35	0.981	0.963	0.949	0.385	0.407	0.610	3.529	3.862	5.607	5.331	4.441	N/A
	CNN-LSTM	47	0.997	0.988	0.964	0.160	0.233	0.516	1.468	2.211	4.742	9.142	5.251	N/A

$R_{c}^{2}$, $R_{v}^{2}$, $R_{t}^{2}$, RMSEC, RMSEV, RMSET, rRMSEC, rRMSEV, rRMSET represent $R^{2}$, RMSE and rRMSE of calibration, validation and test sets, respectively. RPDV and RPDT represent RPD of validation and test sets. LVs are the principal components extracted from PLSR. The optimal number of LVs is determined by the minimum PRESS value obtained during cross-validation, are not applicable to SVM and ELM models (denoted as N/A).

**Table 7:** Evaluation Metrics for Beer Authenticity Classification Model.

Model	PLS-DA					
Method	Full	CARS	SPA	CNN	LSTM	CNN-LSTM
Dimension	228	129	30	27	26	21
ACC_C_	100	100	100	100	100	100
ACC_V_	100	100	100	100	100	100
ACC_T_	98.809	98.809	100	100	100	100
Precision_C_	100	100	100	100	100	100
Precision_V_	100	100	100	100	100	100
Precision_T_	99.074	99.074	100	100	100	100
Recall_C_	100	100	100	100	100	100
Recall_V_	100	100	100	100	100	100
Recall_T_	98.889	98.889	100	100	100	100
Macro F1_C_	100	100	100	100	100	100
Macro F1_V_	100	100	100	100	100	100
Macro F1_V_	98.965	98.965	100	100	100	100
Weighted F1_C_	100	100	100	100	100	100
Weighted F1_V_	100	100	100	100	100	100
Weighted F1_T_	98.808	98.808	100	100	100	100
LVs	18	16	17	13	19	19

ACC_C_, ACC_V_, ACC_T_, Precision_C_, Precision_V_, Precision_T_, Recall_C_, Recall_V_, Recall_T_, Macro F1_C_, Macro F1_V_, Macro F1_T_, Weighted F1_C_, Weighted F1_V_, Weighted F1_T_ represent Accuracy rate, Precision, Recall rate, macro-averaged F1 Score and weighted averaged F1 Score of calibration, validation and test sets, respectively. LVs are the principal components extracted in PLS-DA. The optimal number is determined by the minimum ACC obtained through cross-validation.

## Data Availability

The original contributions presented in the study are included in the article/Supplementary Materials, further inquiries can be directed to the corresponding author.

## Supplementary Materials

The following supporting information can be downloaded at https://www.mdpi.com/article/10.3390/foods14223936/s1; Table S1. Craft Beer and Industrial Beer Sample Information. Table S2. Blended beer sample information. Table S3. CNN Parameter Optimization Range. Table S4. LSTM Parameter Optimization Range. Table S5. CNN-LSTM Parameter Optimization Range. Table S6. Results of Different Spectral Preprocessing Methods for Alcohol Content. Table S7. Results of Different Spectral Preprocessing Methods for Original Wort Concentration. Table S8. Results of Different Spectral Preprocessing Methods for Authenticity Identification.
