# Supplementary material for: Quantitative Detection of Key Parameters and Authenticity Verification for Beer Using Near-Infrared Spectroscopy
Source: Foods. 2025 Nov 17;14(22):3936. doi: 10.3390/foods14223936 (PMC12652515; doi:10.3390/foods14223936)
Supplement: Supplementary file 1 [file foods-14-03936-s001.zip › foods-3981986-supplementary.pdf]

Table S1. Craft Beer and Industrial Beer Sample Information

Serial number	Category	Sample name	Place of Origin	Production Date	Alcohol content (%vol)[a]	Original wort concentration (°P)[a]
1	Craft Beer	Harbin Snow Bear	China	2025/7/10	≥4	11
2	Craft Beer	Feiyero White Beer	China	2025/7/15	≥3.9	11
3	Craft Beer	Tsingtao 1819	China	2025/7/22	≥3.3	9
4	Craft Beer	Cool Little Beer	China	2025/8/9	≥3.6	10
5	Craft Beer	Kersen	China	2025/7/18	≥4.8	12
6	Craft Beer	Polar Brothers	China	2025/7/12	≥3.7	10.5
7	Craft Beer	Caesar Orchard	China	2025/7/20	≥4	11
8	Craft Beer	Caesar King	China	2025/7/14	≥4	11
9	Craft Beer	Crazy Tiger	China	2025/7/21	≥3.6	10
10	Craft Beer	Demaiguan (German Crown)	China	2025/7/11	≥3.6	10
11	Craft Beer	Aigenburg	Germany	2025/7/19	≥4.1	11
12	Craft Beer	Oujia Viper	China	2025/7/16	≥5	12
13	Craft Beer	Snow Bear White Beer	China	2025/7/23	≥3.3	10
14	Craft Beer	Genting Felder	China	2025/7/13	≥3.3	10
15	Craft Beer	Maikelei	China	2025/7/17	≥12	24
16	Craft Beer	Ledingburg Pilsner	China	2025/8/8	≥4.2	11
17	Craft Beer	Lost Coast	United States	2025/7/12	≥8.7	18
18	Craft Beer	Hiccup Beaver (Highway)	United States	2025/7/9	≥7.3	17
19	Craft Beer	Lager Barley	China	2025/7/15	≥4.3	11.5
20	Craft Beer	Hiccup Beaver (Phantom Bride)	China	2025/8/5	≥7.1	15
21	Craft Beer	Zhadan Laopi	China	2025/7/12	≥3.3	9
22	Craft Beer	Fansizhe White Beer	China	2025/7/21	≥4.1	11
23	Craft Beer	Six Swans	China	2025/7/14	≥4.5	13

24	Industrial Beer	Blue Lion Extra Dry	China	2025/7/20	≥4.3	11
25	Industrial Beer	Tsingtao Ice Pure	China	2025/8/3	≥4.0	11
26	Industrial Beer	Harbin Ha Super Fresh	China	2025/7/18	≥3.6	10
27	Industrial Beer	Snowflake Ice Cool	China	2025/7/11	≥3.9	10.3
28	Industrial Beer	Snowflake Pure Draft	China	2025/7/16	≥3.2	8
29	Industrial Beer	Harbin Pure Refresh	China	2025/7/23	≥3.3	10
30	Industrial Beer	1664 White Beer	France	2025/7/11	≥4.8	11.8
31	Industrial Beer	Wusu	China	2025/7/17	≥4.0	11
32	Industrial Beer	Harbin 1900	China	2025/7/8	≥3.6	10
33	Industrial Beer	Tsingtao Pure Draft	China	2025/7/22	≥3.1	8
34	Industrial Beer	Budweiser Pure Draft	China	2025/8/2	≥3.1	8
35	Industrial Beer	Harbin Wheat King	China	2025/7/15	≥3.6	10
36	Industrial Beer	Laoshan Refresh	China	2025/7/19	≥3.1	8

[a] The specified value marked on the beer bottle.

Table S2. Blended beer sample information

Serial number	Auxiliary materials(g)	Ethanol(ml)	Distilled water(ml)	Alcohol content(%vol)	Original wort concentration(°P)
1	23.81	6.32	169.87	3	8
2	23.81	8.42	167.77	4	8
3	23.81	10.53	165.66	5	8
4	23.81	14.74	161.45	7	8
5	26.75	6.32	166.93	3	9
6	26.75	8.42	164.83	4	9
7	26.75	10.53	162.72	5	9
8	26.75	14.74	158.51	7	9
9	29.69	6.32	163.99	3	10
10	29.69	8.42	161.89	4	10
11	29.69	10.53	159.78	5	10
12	29.69	14.74	155.57	7	10
13	32.63	6.32	161.06	3	11
14	32.63	8.42	158.95	4	11
15	32.63	10.53	156.84	5	11
16	32.63	14.74	152.63	7	11
17	35.57	6.32	158.12	3	12
18	35.57	8.42	156.01	4	12
19	35.57	10.53	153.91	5	12
20	35.57	14.74	149.7	7	12

Auxiliary materials refers to the volume of sodium bicarbonate, beer flavorings, and anhydrous citric acid.

Table S3. CNN Parameter Optimization Range

Key Indicators	Parameter	Range
Alcohol content	numResponses	[10, 100]
	InitialLearnRate	[0.001, 0.1]
	L2Regularization	[1e-10, 0.01]
	LearnRateDropFactor	[0.2, 0.9]
Original wort	numResponses	[10, 100]
	InitialLearnRate	[0.001, 0.1]
	L2Regularization	[1e-10, 0.01]
	LearnRateDropFactor	[0.2, 0.9]
Classification and Identification	numResponses	[10, 100]
	InitialLearnRate	[0.001, 0.1]
	L2Regularization	[1e-10, 0.01]
	LearnRateDropFactor	[0.2, 0.9]

numResponses, InitialLearnRate, L2Regularization, and LearnRateDropFactor represent the number of responses in the fully connected layer, the initial learning rate, the L2 regularization factor, and the learning rate drop factor, respectively.

Table S4. LSTM Parameter Optimization Range

Key Indicators	Parameter	Range
Alcohol content	numResponses	[10, 100]
	InitialLearnRate	[0.001, 0.1]
	L2Regularization	[1e-10, 0.01]
	LearnRateDropFactor	[0.2, 0.9]
Original wort	numResponses	[10, 100]
	InitialLearnRate	[0.001, 0.1]
	L2Regularization	[1e-10, 0.01]
	LearnRateDropFactor	[0.2, 0.9]
Classification and Identification	numResponses	[10, 100]
	InitialLearnRate	[0.001, 0.1]
	L2Regularization	[1e-10, 0.01]
	LearnRateDropFactor	[0.2, 0.9]

numResponses, InitialLearnRate, L2Regularization, and LearnRateDropFactor represent the number of responses in the fully connected layer, the initial learning rate, the L2 regularization factor, and the learning rate drop factor, respectively.

Table S5. CNN-LSTM Parameter Optimization Range

Key Indicators	Parameter	Range
Alcohol content	numResponses	[10, 100]
	FiltSize	[5, 15]
	numChannels	[16, 40]
	MaxEpochs	[50, 400]
	numHiddenUnits	[30, 100]
	InitialLearnRate	[0.001, 0.1]
	LearnRateDropPeriod	[50, 150]
	L2Regularization	[1e-10, 0.01]
	LearnRateDropFactor	[0.2, 0.9]
Original wort	numResponses	[10, 100]
	FiltSize	[5, 15]
	numChannels	[16, 40]
	MaxEpochs	[50, 400]
	numHiddenUnits	[40, 60]
	InitialLearnRate	[0.001, 0.1]
	LearnRateDropPeriod	[50, 150]
	L2Regularization	[1e-10, 0.01]
	LearnRateDropFactor	[0.2, 0.9]
Classification and Identification	numResponses	[10, 100]
	FiltSize	[5, 15]
	numChannels	[16, 40]
	MaxEpochs	[50, 400]
	numHiddenUnits	[40, 60]
	InitialLearnRate	[0.001, 0.1]
	LearnRateDropPeriod	[50, 150]
	L2Regularization	[1e-10, 0.01]
	LearnRateDropFactor	[0.2, 0.9]

numResponses, FiltSize, numChannels, MaxEpochs, numHiddenUnits, InitialLearnRate, LearnRateDropPeriod, L2Regularization, and LearnRateDropFactor represent the number of responses in the fully connected layer, the size of the convolutional kernel, the number of convolutional channels, the maximum number of iterations, the number of hidden units in the LSTM, the initial learning rate, the learning rate drop period, the L2 regularization factor, and the learning rate drop factor, respectively.

Table S6. Results of Different Spectral Preprocessing Methods for Alcohol Content

Preprocessing methods	R_{cv}^2	RMSECV(%)	RPDCV	LVs
N/A	0.939	0.358	4.685	10
SG	0.939	0.358	4.682	10
MSC	0.931	0.379	4.447	13
SNV	0.929	0.386	4.374	13
WD	0.939	0.357	4.702	10
FTD	0.939	0.358	4.699	10
SG+MSC	0.925	0.389	4.432	12
SG+SNV	0.929	0.380	4.496	13
SG+WD	0.939	0.359	4.676	10
SG+FTD	0.939	0.358	4.700	10
MSC+SNV	0.929	0.383	4.384	13
MSC+WD	0.935	0.366	4.630	13
MSC+FTD	0.920	0.402	4.309	9
SNV+WD	0.936	0.363	4.649	14
SNV+FTD	0.921	0.401	4.317	9
WD+FTD	0.939	0.358	4.676	10

R_{cv}^2 、RMSECV and RPDCV represent R^2 、RMSE and RPD of cross-validation, respectively. LVs are the principal components extracted from PLSR. The optimal number of LVs is determined by the minimum PRESS value obtained during cross-validation.

Table S7. Results of Different Spectral Preprocessing Methods for Original Wort Concentration

Preprocessing methods	R_{cv}^2	RMSECV(%)	RPDCV	LVs
N/A	0.682	1.208	2.129	12
SG	0.686	1.206	2.141	12
MSC	0.713	1.160	2.217	10
SNV	0.780	1.016	2.506	11
WD	0.747	1.077	2.382	13
FTD	0.670	1.227	2.119	12
SG+MSC	0.710	1.146	2.256	12
SG+SNV	0.757	1.065	2.433	11
SG+WD	0.687	1.197	2.160	12
SG+FTD	0.676	1.220	2.118	12
MSC+SNV	0.775	1.019	2.489	11
MSC+WD	0.687	1.178	2.217	10
MSC+FTD	0.681	1.172	2.254	14
SNV+WD	0.721	1.119	2.319	10
SNV+FTD	0.720	1.120	2.336	12
WD+FTD	0.710	1.162	2.220	12

R_{cv}^2 、RMSECV and RPDCV represent R^2 、RMSE and RPD of cross-validation, respectively. The optimal number of LVs is determined by the minimum PRESS value obtained during cross-validation.

Table S8. Results of Different Spectral Preprocessing Methods for Authenticity Identification

Preprocessing methods	ACCCV(%)	PrecisionCV(%)	RecallCV(%)	LVs
N/A	99.440	99.380	99.490	15
SG	99.180	99.160	99.260	15
MSC	99.340	99.350	99.370	18
SNV	99.320	99.330	99.350	19
WD	99.570	99.610	99.600	16
FTD	99.390	99.390	99.460	18
SG+MSC	99.130	99.180	99.170	17
SG+SNV	99.110	99.170	99.150	17
SG+WD	99.400	99.410	99.440	15
SG+FTD	99.380	99.380	99.450	17
MSC+SNV	99.110	99.180	99.040	12
MSC+WD	99.120	99.180	99.160	18
MSC+FTD	99.080	99.140	99.020	16
SNV+WD	99.240	99.280	99.270	16
SNV+FTD	99.150	99.210	99.080	15
WD+FTD	99.580	99.640	99.620	19

ACCCV、PrecisionCV and RecallCV represent ACC、Precision and Recall of cross-validation, respectively. LVs are the principal components extracted in PLS-DA. The optimal number is determined by the minimum ACC obtained through cross-validation.
